# Supplementary material for: Microstructure, Mechanical Properties and Fracture Behavior of Micron-Sized TiB2/AlZnMgCu(Sc,Zr) Composites Fabricated by Selective Laser Melting
Source: Materials (Basel). 2023 Mar 6;16(5):2112. doi: 10.3390/ma16052112 (PMC10003815; doi:10.3390/ma16052112)
Supplement: Supplementary file 1 [file materials-16-02112-s001.zip › materials-2238846-supplementary.pdf]

Supplementary Infomation

Microstructure, mechanical properties and fracture behavior of micron-sized TiB_2/AlZnMgCu(Sc,Zr) composites fabricated by selective laser melting

Peng Yin[1,2,3], Yongzhong Zhang[1,2,3]*, Yantao Liu[1,2,3], Wei Pan[1,2,3], Zhuoheng Liang[1,2,3], Shuobing Shao[1,2,3]

1 National Engineering & Technology Research Center for Non-Ferrous Metals Composites, GRINM Group Corporation Limited, Beijing 101407, China

2 Grinm Metal Composites Technology Co., Ltd., Beijing 101407, China

3 General Research Institute for Nonferrous Metals, Beijing 100088, China

* Correspondence: yyzhang@grinm.com (Y. Z.); Tel.: +86-13501008631(Y. Z.)

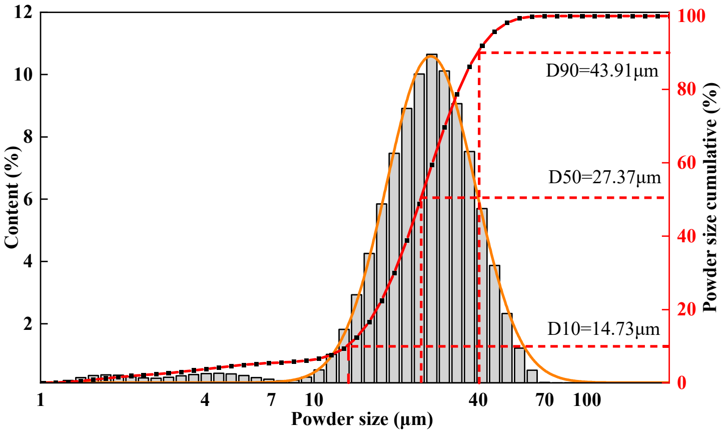

Supplementary Figure S1. Particle size distribution of TiB2/AlZnMgCu(Sc,Zr) powder, D10, D50 and D90 are 14.73 μm, 27.37 μm and 43.91 μm, respectively

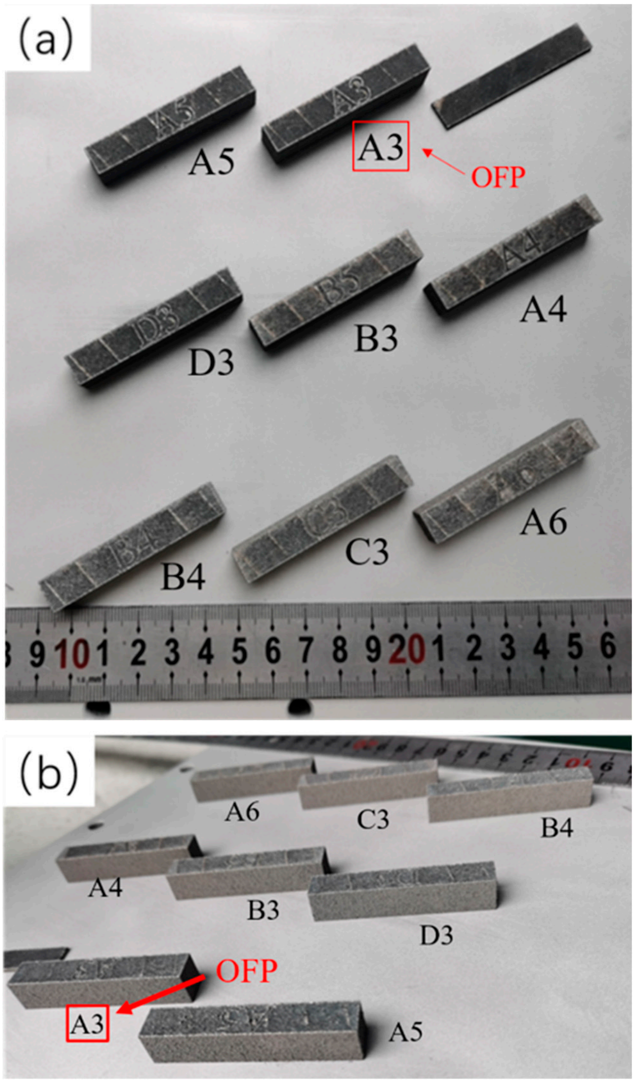

Supplementary Figure S2. The horizontal samples formed by SLM (a) upper surface and (b) side surface

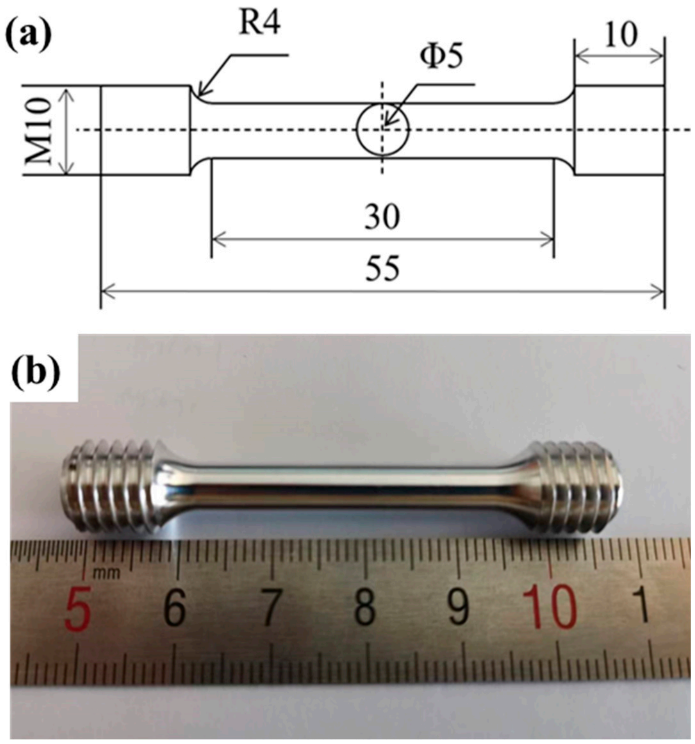

Supplementary Figure S3. Diagram of horizontal tensile bar (a) schematic diagram (mm) and (b) real sample

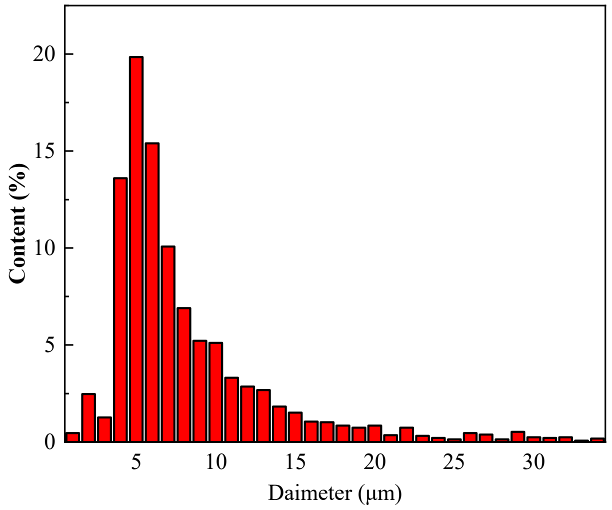

Supplementary Figure S4. Pore size distribution of TiB_2/AlZnMgCu(Sc,Zr) fabricated by SLM.

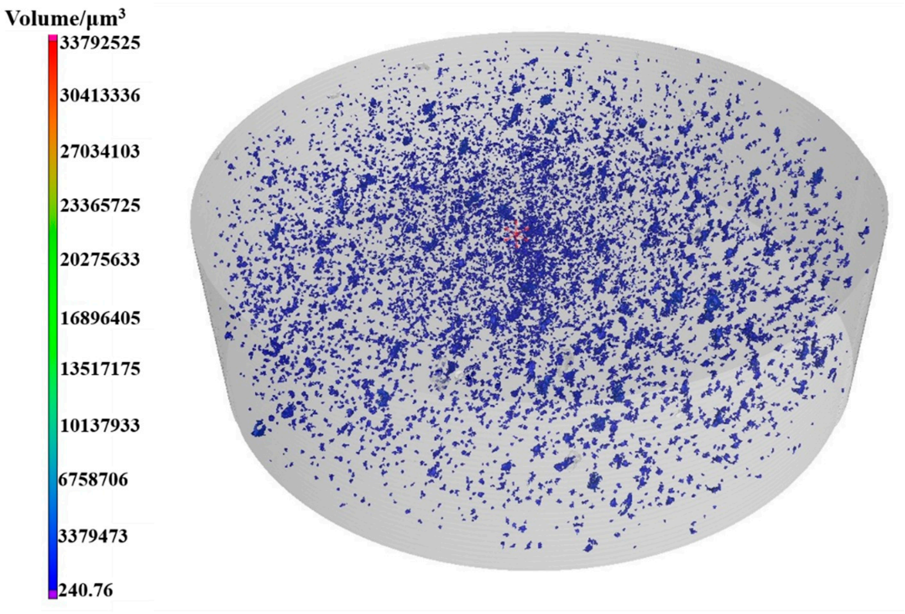

Supplementary Figure S5. Distribution of micropores in TiB_2/AlZnMgCu(Sc,Zr) composite samples

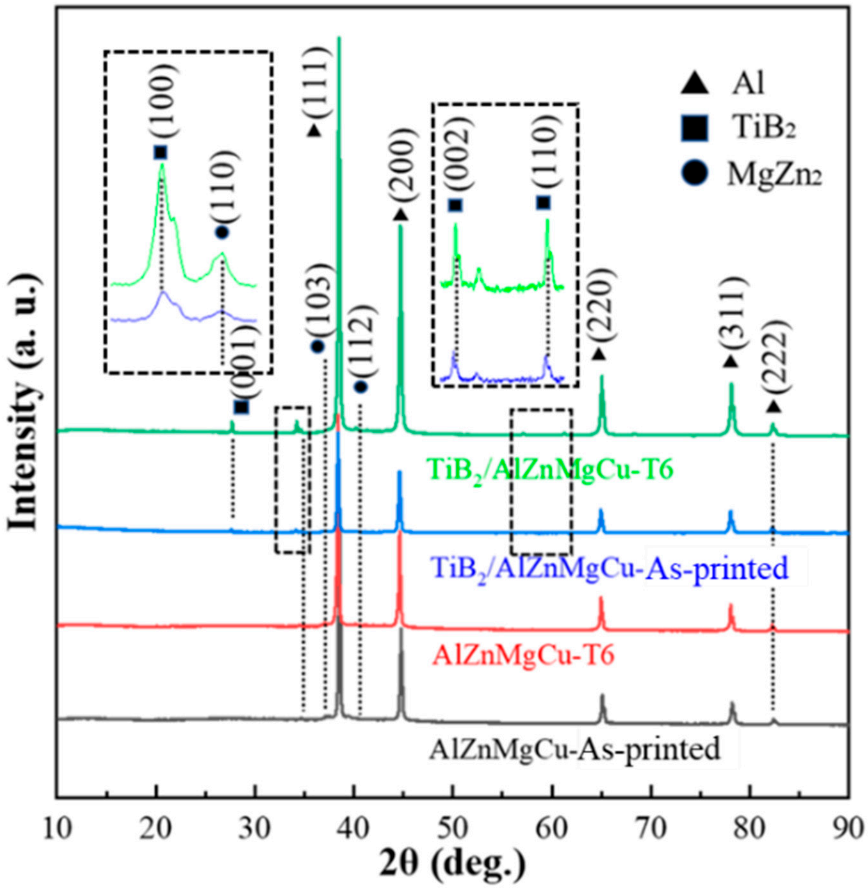

Supplementary Figure S6. Phase analysis of AlZnMgCu(Sc,Zr) alloy and TiB2/AlZnMgCu(Sc,Zr) composite before and after heat treatment

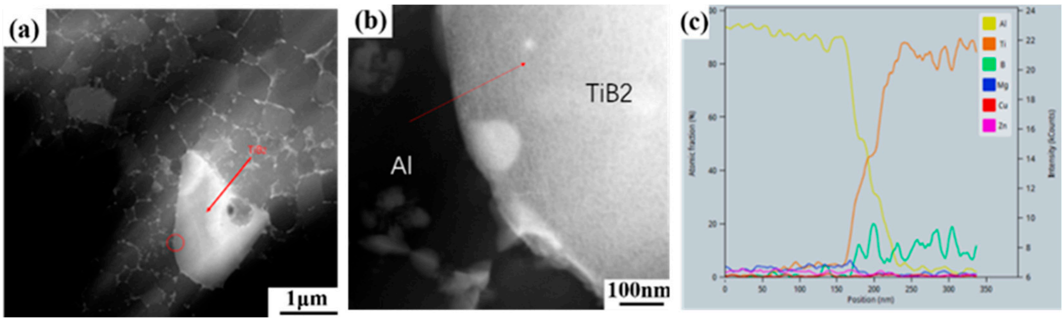

Supplementary Figure S7. HAADF image of the interface between micron-TiB_2 particle and Al matrix in SLM-fabricated TiB_2/AlZnMgCu(Sc,Zr) composite (a) in relatively low magnification, (b) in relatively high magnification and (c) EDS liner scanning

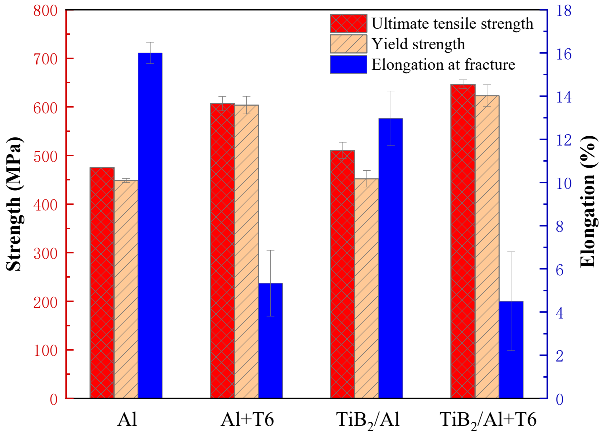

Supplementary Figure S8. Comparison of mechanical properties of AlZnMgCu(Sc,Zr) and TiB_2/AlZnMgCu(Sc,Zr) composite before and after heat treatment

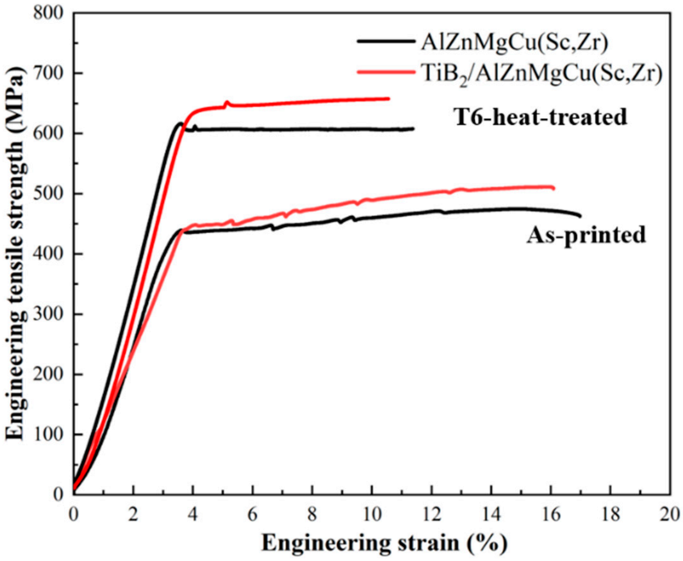

Supplementary Figure S9. Tensile engineering stress-strain curves of TiB_2/AlZnMgCu(Sc,Zr) composite and AlZnMgCu(Sc,Zr) alloy samples before and after heat treatment
